# Research on the Relationship between Cutting Force and Machined Surface Quality in Micro Ball End-Milling of Potassium Dihydrogen Phosphate Crystal

**DOI:** 10.3390/mi9110574

**Published:** 2018-11-05

**Authors:** Ni Chen, Chunya Wu, Mingjun Chen, Liang Li, Ning He

**Affiliations:** 1 College of Mechanical and Electrical Engineering, Nanjing University of Aeronautics and Astronautics, Nanjing 210016, China; liliang@nuaa.edu.cn (L.L.); drnhe@nuaa.edu.cn (N.H.); 2 Center for Precision Engineering, Harbin Institute of Technology, Harbin 150001, China; wuchunya1982@163.com

**Keywords:** KDP crystal, micro ball end-milling, cutting force, FFT analysis, machined surface quality

## Abstract

Potassium dihydrogen phosphate (KDP or KH_2_PO_4_) crystal is widely used as terminal frequency converters in inertial confinement fusion (ICF). However, KDP crystal is a typical difficult-to-cut optical crystal with the characteristic of soft-brittle. In this work, the relationship between cutting force and processed surface quality in micro ball end-milling of KDP crystal with various depth of cut and spindle speed is studied by carried out the micro-milling experiments. Fast Fourier Transform (FFT) algorithm is used to diagnose the recorded cutting force. The periodic change of cutting force and the cutting force after filtering noises can be got through FFT analysis. Through calculating the correlation coefficients between the static component of thrust force and roughness value Ra of machined grooves, as well as the peak-valley (P-V) value of thrust force and dimensional error of machined grooves, the roughness value Ra and dimensional error of machined grooves would be predicted by monitoring the static component and P-V value of the thrust force, respectively. The relatively large spindle speed helps to reduce the roughness value Ra. The spindle speed with moderate value is recommended to reduce the dimensional error of machined groove because the dimensional error of machined groove will increase when the spindle speed is small enough (causing brittle cutting) or large enough (reducing cutting stability).

## 1. Introduction

In inertial confinement fusion (ICF), potassium dihydrogen phosphate (KDP or KH_2_PO_4_) crystal is widely used as optical Pockels-cells and terminal frequency converters, due to its large electro-optic coefficient and good quality of growth characteristics [1]. Considering that micro ball end-milling has several principal advantages over other available micro-fabrication technologies such as abilities to fabricate complex 3D geometries with high aspect ratio in a wide variety of metallic and non-metallic materials [2,3,4], it has been widely regarded as a useful method to repair micro-defects, which is generated on the surface of KDP crystal during laser target shooting or machining [5,6].

KDP crystal is a typical difficult-to-cut optical crystal with the characteristic of soft-brittle, therefore the brittle cutting occurs and micro scallops appear on the processed surface easily [7,8,9,10]. Therefore, the ductile cutting of KDP crystal in micro-machining is researched by many experts. Chen et al. [11] obtained the critical undeformed chip thickness for ductile cutting in flying cutting of KDP crystal by theoretical deduction of cutting principle and received ultra-smooth surface with the roughness Ra value of 5.1 nm. Tie et al. [12] researched the turning of KDP crystal and discovered that the critical undeformed chip thickness for brittle-ductile transition varies from about 200 nm to 1000 nm with the change of cutting directions. The roughness Ra value on the whole machined surface which is less than 2 nm can be got. Recently, Chen et al. [13,14] revealed that the surface quality is closely related with the cutting parameters and the surface roughness value Ra of machined grooves could be controlled below 20 nm by optimizing the cutting parameters both in micro milling of KDP crystal.

Moreover, many researchers discovered that size effect, which is manifested as ploughing effect is appeared usually in micro machining of metals [15,16] and several researchers (like Arif et al. [17] and Amin et al. [18]) found size effect in end-milling of brittle materials. In particular, Chen et al. [14] found that size effect which is manifested as severe ploughing and rubbing effect is appeared when the undeformed chip thickness is smaller than a certain value in micro milling of KDP crystal. However, the above researchers mainly studied how cutting parameters influences the machined surface roughness and few researchers studied which factors got easily in online machining would be used to monitor the machined surface roughness in micro machining of KDP crystal. Moreover, the dimensional accuracy of machined KDP crystal surface has great influence on the optical transmission characteristic of KDP crystal [19,20]. Few researchers studied the dimensional accuracy of machined surface in micro machining of KDP crystal.

In micro machining of metals, several researchers have studied how cutting parameters influence the cutting force and machined surface quality. Sooraj and Mathew [21] studied the tangential and radial components of cutting forces, specific forces and roughness value of processed surface in micro end-milling of brass and found that the drastic variation of specific cutting force and the cutting force clearly indicate the size effect and the change of machined surface roughness in micro machining. Wang et al. [22] studied the cutting force in reaming of ZL102 aluminum cast alloys and found that the amplitude of fundamental frequency for cutting force is closely related with the cylindricity of reamed holes. Fard and Bordatchev [23] experimentally investigated the effect of tool orientation on the cutting force, final surface geometry and quality in five-axis micro ball end-milling of brass and found that the tilt angle has great influence on the cutting force and machined surface quality; moreover, the reduced value of cutting forces in inclined machining contributed to the improved surface quality for the considered cases. Above researchers found that the cutting force is connected to the machined surface quality in micro machining of metals.

In this paper, Fast Fourier Transform (FFT) algorithm is used to diagnose the recorded cutting force in micro ball end-milling of KDP crystal. The FFT analysis is also used to study the periodicity of cutting force and filter the noises out of the cutting force. In order to find an effective way to monitor the processed surface quality in micro ball end-milling of KDP crystal, the relationship between processed surface quality and cutting force will be researched in detail. Then, the recommended cutting parameters will be given out.

## 2. Experimental Procedure

### 2.1. The Experimental Set-Up

**Figure 1:**
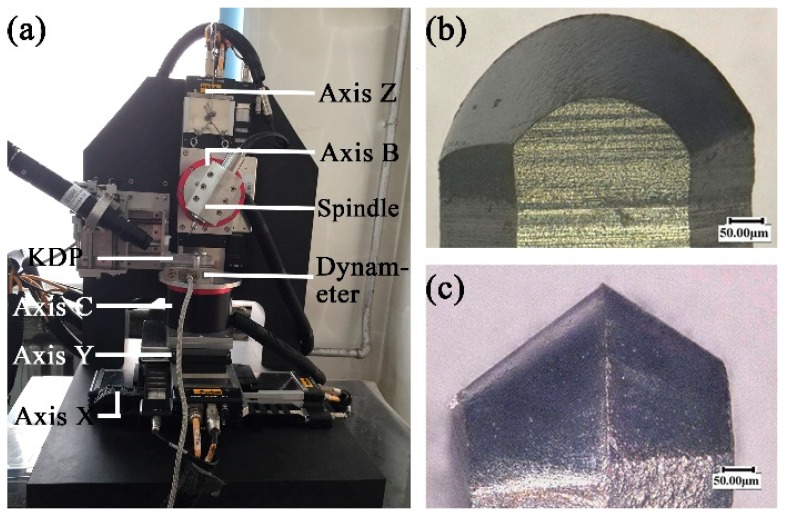
The machine tool system: (**a**) main components; (**b**,**c**) side views of polycrystalline diamond (PCD) micro ball end mill.

The home-made five-axis machine tool is used to carry out the micro milling experiments. The machine tool mainly includes three line axes of X Y Z with the positional precision of ±0.35 μm/10 mm and resolution of 0.1 μm and two rotational axes of B C. A NSK^®^ electrical spindle (NSK Ltd., Tokyo, Japan) with the run-out of less than 1 μm is used in the machine tool. The experimental platform is illustrated in Figure 1a. The self-fabricated polycrystalline diamond (PCD) micro ball end mill with the diameter of 500 μm is used in the experiments, as shown in Figure 1b,c. The rake angle and relief angle of PCD micro ball end mill are −50° and 40°, respectively. The workpiece of KDP crystal with dimension of 95 mm × 27 mm × 14 mm is used in the experiments.

### 2.2. The Design of Cutting Experiments

In order to study the relationship between cutting force and machined surface quality under different cutting parameters, the spindle speed is changed from 10,000 to 70,000 r/min with an interval of 10,000 r/min. The feed per tooth is arranged as 1 μm/z and depth of cut is arranged as 2, 5 and 8 μm. The detailed cutting parameters are illustrated in Table 1. In order to avoid the lowest point of the ball mill touching and deteriorating the processed surface, the tilt angle of 30° is set for the ball mill in the experiments.

### 2.3. Cutting Force Measurement and FFT Analysis

The dynamometer (Kistler^®^ 9256C2, Kistler, Winterthur, Switzerland) is used to measure the cutting force in the experiments. The screws are used to fix the workpiece of KDP crystal on the dynamometer and the dynamometer on the worktable. The machining forces is recorded at a sampling frequency of 30,000 Hz. Fast Fourier Transform (FFT) algorithm is used to diagnose the recorded cutting force. The periodic change of cutting force and the filtering of noises in the cutting force are studied through FFT analysis.

### 2.4. Surface Detection and Measurement Method of Surface Roughness

The VHX-1000E, an 3-dimensional microscopy (Keyence, Osaka, Japan) is used to record the machined surface morphology. The roughness value Ra of micro grooves is detected by PGI1240 contour graph (Taylor Hobson, Leicester, UK). The tip radius used in PGI 1240 is 2 μm. The rate of closure chooses the cut-off wavelength of 0.0025 mm. After the original contour data is obtained, the surface shape is removed by random software. For each micro groove, five different areas are detected. The mean value of these measured values is taken as the machined surface roughness Ra value, while the standard deviation of these measured values is taken as the detecting error.

### 2.5. Measurement of Dimensional Error of Machined Groove Width

**Figure 2:**
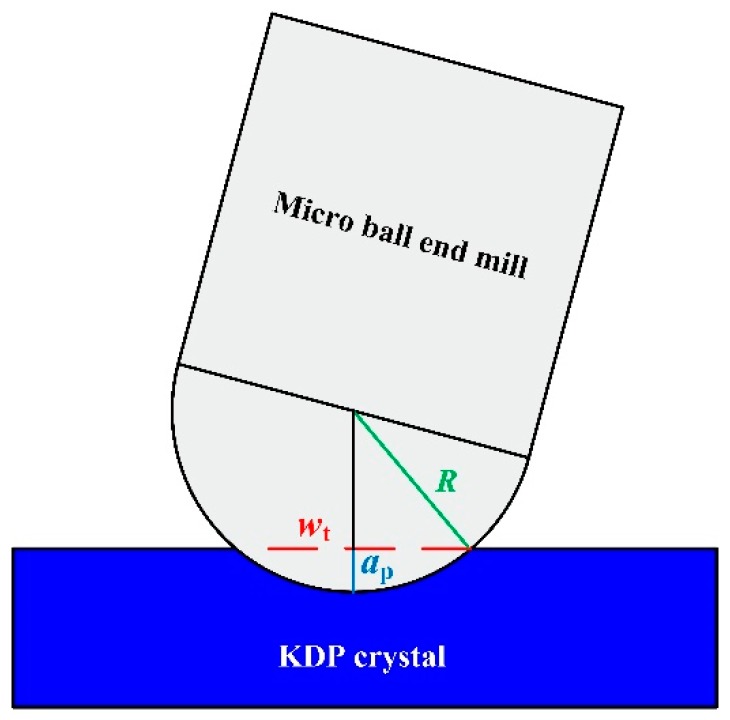
The diagram of micro ball end-milling process.

The machined groove width is also measured by VHX-1000E (Keyence^®^) and five different areas on the groove are measured. The theoretical machined groove width is shown in Figure 2 and it can be calculated by Equation (1). The mean and standard deviation of subtraction values between measured and theoretical machined groove width are taken as dimensional error of machined groove width and the detecting error respectively. The calculation of dimensional error of machined groove and detecting error are shown in Equations (2) and (3).

(1) $$w_{t}=\sqrt{R^{2}-{\left(R-a_{p}\right)}^{2}}\times 2$$

where *w*_t_ is the theoretical machined groove width (μm), *R* is the radius of micro ball end mill and *a*_p_ is the depth of cut in micro ball end-milling (μm).

(2) $$w_{e}=\sqrt{\frac{\sum_{i=1}^{5}\left|w_{\mathrm{mi}}-w_{t}\right|}{5}}$$

where *w*_m_ is the measured machined groove width (μm) and *w*_e_ is the dimensional error of machined groove width (μm).

(3) $$w_{\mathrm{de}}=\sqrt{\frac{\sum_{i=1}^{5}{\left(\left|w_{\mathrm{mi}}-w_{t}\right|-w_{e}\right)}^{2}}{5}}$$

where *w*_de_ is the detecting error for dimensional error of machined groove width (μm).

## 3. Results and Discussion

### 3.1. FFT Analysis of the Signals of Cutting Force

At the feed speed of 1 μm/z, depth of cut of 2 μm and spindle speed of 6 × 10^4^ r/min, the micro grooves are processed by micro PCD ball end mill and the three-dimensional cutting force under this cutting parameter is measured by the dynamometer. In micro milling of KDP crystal, the cutting force should have a certain periodicity because the cutting thickness in next cutting cycle is same as in current cutting cycle when the cutting parameter is fixed, regardless of the minimum value for undeformed chip thickness. The fundamental frequency *f*_0_ for cutting force can be expressed in Equation (4):

(4) $$f_{0}=\frac{1}{T}=\frac{1}{\frac{60}{n}}=\frac{n}{60}$$

where *n* is the spindle speed (r/min) and *T* is the time per revolution (s).

It is well known that the fundamental frequency is only closely related with spindle speed (*n*). When *n* = 60,000 r/min is substituted in Equation (4), *f*_0_ is equal to 1000 Hz. The perform frequency spectrum analysis is carried out to research the source of cutting force vibration and its periodicity in detail.

**Figure 3:**
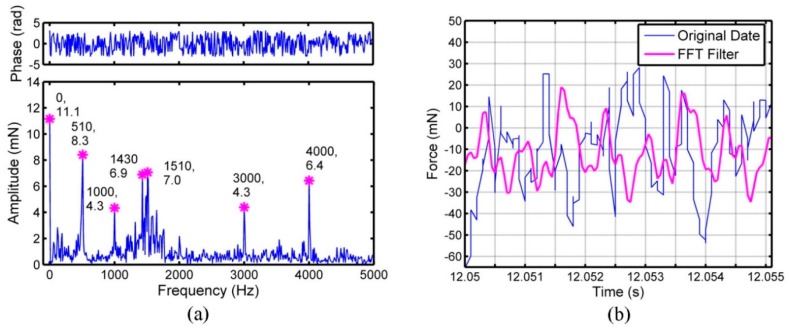
The cutting force signals of feed direction (*F*_x_): (**a**) Fast Fourier Transform (FFT) analysis on feed force, (**b**) comparative curves between original data and data after FFT filtering.

**Figure 4:**
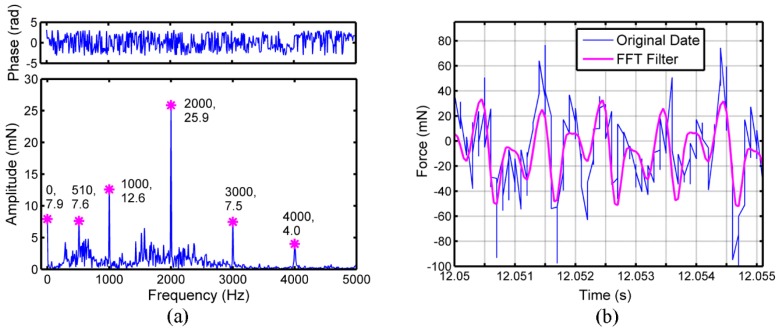
The cutting force signals of cross feed direction (*F*_y_): (**a**) FFT analysis on cross feed force, (**b**) comparative curves between original data and data after FFT filtering.

**Figure 5:**
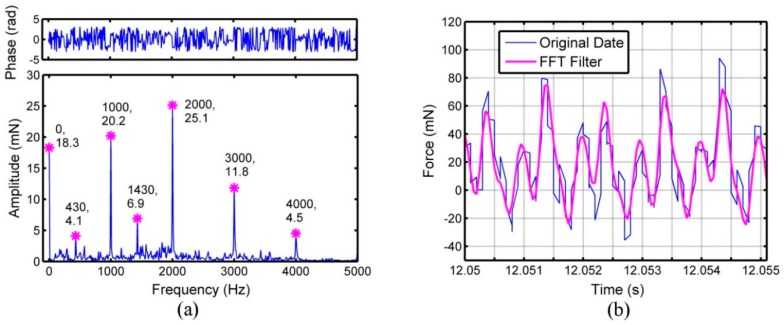
The cutting force signals of thrust direction (*F*_z_): (**a**) FFT analysis on thrust force, (**b**) comparative curves between original data and data after FFT filtering.

The FFT results for feed force (*F*_x_), cross feed force (*F*_y_) and thrust force (*F*_z_) are displayed in Figure 3a, Figure 4a and Figure 5a, respectively. The feed force, cross feed force and thrust force mainly consist of several sub-force components with the characteristic frequencies, which are marked in Figure 3a, Figure 4a and Figure 5a. Besides the frequency of 0 Hz, the sub-force with the frequency of 510 Hz occupies the largest proportion in the original feed force and the sub-force components with the frequencies of 1000, 1430, 1510, 3000 and 4000 Hz also have significant proportions. For original cross feed force and thrust force, the sub-force with the frequency of 2000 Hz occupies the largest proportion. The reason is that the micro ball end mill with double-tooth is used leading to two times of fundamental frequency. The sub-force components with the frequencies of 510 Hz, 1000 Hz, 3000 Hz and 4000 Hz also have significant proportions in original cross feed force, while the sub-force components with the frequencies of 430, 1000, 1430, 3000 and 4000 Hz have significant proportions in original thrust force. The characteristic frequencies of three-dimensional cutting forces should be an integral multiple of the fundamental frequency of 1000 Hz [22], however, the characteristic frequencies of 430, 510, 1430 and 1510 Hz are appeared in the cutting force which may be caused mainly by the minimum cutting thickness effect [21]. As shown in reference [14], the minimum value for maximum undeformed chip thickness in one cutting circle to form the chips is more likely between 238.9 and 339.9 nm in micro ball end milling of KDP crystal and the feed per tooth is between 1.16 μm/z and 1.66 μm/z under the depth of cut of 2 μm. In this work, the feed per tooth of 1 μm/z is set, therefore the chips are formed under one or two tool passes. The cutting force under the condition that the chips formed on first tool pass and on second tool pass is different. So, there will exist the characteristic frequencies of cutting force around 500 Hz. The characteristic frequencies of 1430 and 1510 Hz are the conjoint results of minimum chip thickness effect and spindle speed.

The expression for feed force, cross feed force and thrust force can be written as the form of Fourier series:

(5a) $$F_{x}=A_{\mathrm{nx}}\cos(w_{\mathrm{nx}}t+\varphi_{\mathrm{nx}})$$

(5b) $$F_{y}=A_{\mathrm{ny}}\cos(w_{\mathrm{ny}}t+\varphi_{\mathrm{ny}})$$

(5c) $$F_{z}=A_{\mathrm{nz}}\cos(w_{\mathrm{nz}}t+\varphi_{\mathrm{nz}})$$

where *A*_n_, *ω*_n_ and *φ*_n_ are the amplitude, frequency and phase of the force component respectively.

The feed force, cross feed force and thrust force obtained through the Equation (5), can be regarded as the cutting forces after filtering noises. The comparisons between the original three-direction cutting forces and the cutting forces after filtering are performed in Figure 3b, Figure 4b and Figure 5b. The magnitude of vibration for three-dimensional cutting forces after FFT filtering is smaller than original emission signals. The original cross feed force and thrust force match well with the data after FFT filtering, while the original feed force has some difference with the data after FFT filtering. Among three-dimensional cutting forces, the difference of amplitude between the original cutting force and the cutting force after FFT filtering is largest in feed force, while smallest for thrust force. The reason is that the measurement of feed force is influenced easily by the noises from the external environment because the feed force is so small [24].

### 3.2. The Relationship between Cutting Force and Machined Surface Quality

The cutting force can be decomposed into three components generally-static, quasi-dynamic and dynamic. The static cutting force is mainly manifested by a mean value of the cutting force. A peak-valley (P-V) value, which is the mean difference value between maximum and minimum cutting forces within a spindle revolution, is one of the commonly used parameters for estimating a quasi-dynamic component of the cutting force [23]. The dynamic component of the cutting force is usually due to the cutting process dynamics and can be considered as a random value. It will be filtered by the FFT method. Since the feed force is relatively small and irregular when compared with other two components and the measurement of feed force is influenced easily by the noises from the external environment, only the thrust force and cross feed force are studied later.

#### 3.2.1. The Relationship between the Static Cutting Force and Roughness Value Ra of Machined Surface

**Figure 6:**
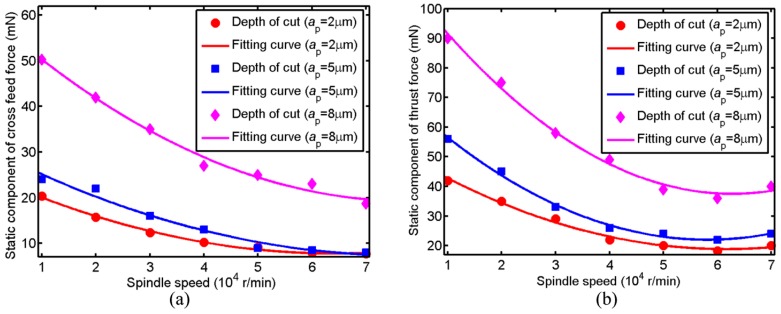
The change of static component of cutting force with spindle speed under different depth of cut (**a**) the static component of cross feed force; (**b**) the static component of thrust force.

The cutting force at 0 Hz in FFT analysis represents the mean value of cutting force. Thus, the cutting force at 0 Hz in FFT analysis is used as the static cutting force. The date of cutting force which is over 100 revolutions at the stable machining is used to carry out the FFT analysis. Figure 6 shows the change of static component of the cross feed force and thrust force with spindle speed under different depth of cut. For these three depth of cut, the static component of the cross feed force decreases with the increase of spindle speed from 10,000 to 70,000 r/min. The reason is that thermal softening effect is more dominated that strain-hardening effect leading to the increase of the ductility with the increase of spindle speed in micro ball end-milling of KDP crystal [14]. Relatively speaking, the static component of the cross feed force decreases sharply with the spindle speed increasing from 10,000 to 40,000 r/min, this is mainly caused by the brittle cutting when spindle speed is at a relatively small value. The static component of the cross feed force grows with the increase of depth of cut and its difference under depth of cut of 2 and 5 μm is very small. The reason is that the severe size effect is existed at the depth of cut of 2 increasing the cross feed force [14]. The static component of the thrust force decreases with the increase of spindle speed ranging from 10,000 to 60,000 r/min, while it increases with the increase of spindle speed ranging from 60,000 to 70,000 r/min. The changing trend of the static component of the thrust force is similar to the static component of the cross feed force and the static component of the thrust force is two times larger than the static component of the cross feed force.

**Figure 7:**
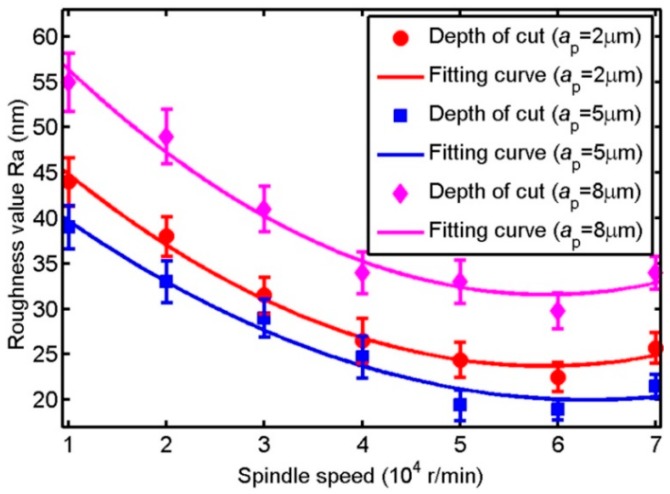
The change of roughness value Ra of machined grooves with spindle speed under different depth of cut.

The change of roughness value Ra of machined grooves with spindle speed under different depth of cut shows in Figure 7. Under depth of cut of 2, 5 and 8 μm, the roughness value Ra of machined grooves is decreases with the augment of spindle speed ranging from 10,000 to 60,000 r/min, while it increases with the augment of spindle speed ranging from 60,000 to 70,000 r/min and its changing trend is very similar to the static component of the thrust force. However, the roughness value Ra of machined grooves decreases to a lowest value at the depth of cut of 5 μm when the depth of cut increase from 2 μm to 8 μm and the roughness value Ra of machined grooves is larger when the depth of cut is at 2 μm and 8 μm. This is mainly caused by the size effect (ploughing effect) in micro milling of KDP crystal. As stated in our previous paper [14], due to the size effect, critical value for maximum undeformed chip thickness in one cutting process to generate the chips is around the value between 238.9 and 339.9 nm. The roughness value Ra of machined grooves decreases with the maximum thickness increasing to 238.9 nm and reaching lowest value when maximum undeformed chip thickness at the range of 238.9 and 339.9 nm, then roughness value Ra turns to increase with the augment of maximum undeformed chip thickness. In this paper, under the feed per tooth of 1 μm/z, the maximum undeformed chip thickness under one cutting process is 206.5, 299.9 and 356.7 nm when depth of cut is 2, 5 and 8 μm, respectively.

The changing trend of roughness value Ra of machined grooves and the static component of thrust force is similar except for the size effect area. The correlation coefficients between the static component of thrust force and roughness value Ra of machined grooves with spindle speed under different depth of cut are calculated out using the function of *corrcoef* in Matlab^®^ (2011a, Mathworks Inc., Natick, MA, USA) as shown in Table 2. The correlation coefficients are larger than 0.97 under these three depth of cut indicating the close relationship between the static component of thrust force and roughness value Ra of machined grooves. Therefore, adding the size effect study in our previous paper [14], the roughness value Ra of machined grooves would be predicted by monitoring the static component of the thrust force.

#### 3.2.2. The Relationship between P-to-V Value of Cutting Force and Dimensional Error of Machined Groove

The tool vibration has an unfavorable effect on processing quality, especially, dimensional accuracy of machined surface and it is highly determined by the vibration of cutting force. It is widely recognized that the P-to-V (P-V) value of the cutting force could represent the vibration of cutting force [21,23]. The cutting forces over 100 revolutions are analyzed to get the P-V values of the cutting force.

**Figure 8:**
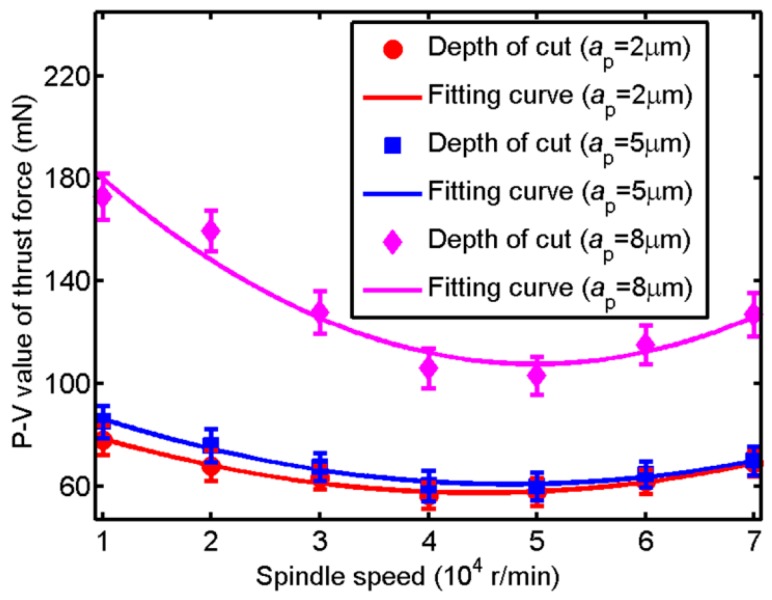
The change of P-V value of thrust force with spindle speed under different depth of cut.

The change of P-V value of thrust force with spindle speed under different depth of cut is shown in Figure 8. At all depth of cut, the P-V value of thrust force decreases with the augment of spindle speed ranging from 10,000 to 40,000 r/min and it has a minimum value when spindle speed around 40,000 and 50,000 r/min, while it increases with the augment of spindle speed ranging from 50,000 to 70,000 r/min. Therefore, the vibration of thrust force decreases with the augment of spindle speed and then it increases. The reasons is that the appearance of brittle cutting leads to the high vibration of thrust force when spindle speed is at a relatively small value, while reduction of cutting stability leads to the high vibration of thrust force when spindle speed is at a relatively large value.

**Figure 9:**
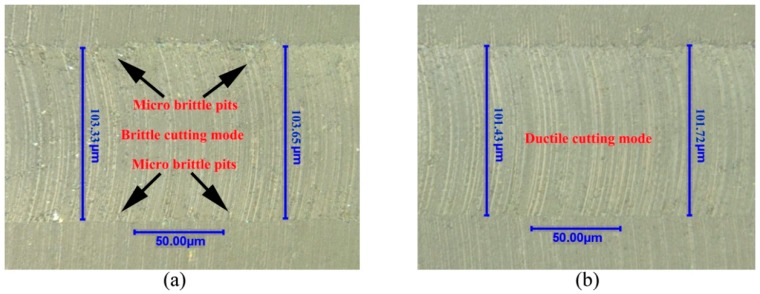
The morphology of machined grooves under the depth of cut of 5 μm with the spindle speed of: (**a**) 10,000 r/min, (**b**) 40,000 r/min.

**Figure 10:**
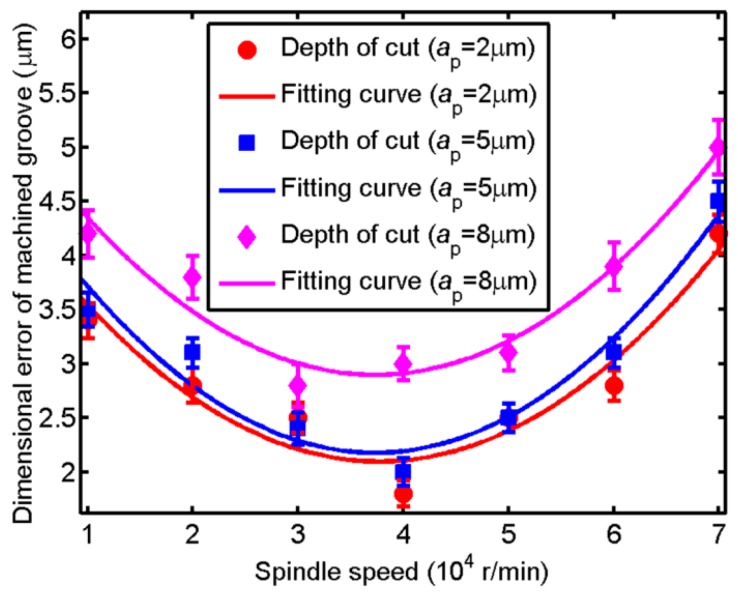
The change of dimensional error of machined groove with spindle speed under different depth of cut.

In micro ball end-milling, dimensional error of machined surface is closely related with the performance of workpiece. Taking the machined groove at the depth of cut of 5 μm as an example, Figure 9 shows the groove width at the spindle speed of 10,000 to 40,000 r/min under the depth of cut of 5 μm, respectively. The theoretical groove width at the depth of cut of 5 μm which can be calculated by Equation (1) is 99.50 μm. As shown in Figure 9a, the groove widths at two different areas are 103.33 and 103.65 μm, respectively. The brittle cutting mode, as shown in Figure 9a, which causing the vibration of cutting process leads to large dimensional error of machined groove (around 3.5 μm). The dimensional error of machined groove is smallest at the spindle speed of 40,000 r/min and the surface quality of machined groove is perfect with regular tool marking indicating ductile cutting, as shown in Figure 9b. The dimensional error of machined groove which is represented by dimensional error of groove width is studied. Figure 10 shows the change of dimensional error of machined groove with spindle speed under different depth of cut. The change trend of dimensional error of machined groove is almost same with the P-V value of thrust force. The minimal value of dimensional error of machined groove is achieved when the spindle speed is around 40,000 r/min and the maximum value of dimensional error of machined groove is achieved when the spindle speed is around 70,000 r/min, meanwhile, the dimensional error of machined groove also has a relatively large value when the spindle speed is around 10,000 r/min.

The correlation coefficients between the P-V value of thrust force and dimensional error of machined grooves with spindle speed under different depth of cut are calculated out using the function of *corrcoef* in Matlab^®^, as shown in Table 3. The correlation coefficients are larger than 0.51 under these three depth of cut indicating the relatively close relationship between the P-V value of thrust force and dimensional error of machined grooves. Therefore, the dimensional error of machined groove would be predicted by monitoring the P-V value of thrust force indirectly.

## 4. Conclusions

This study investigates in depth the relationship between cutting force and quality of machined groove in micro ball end-milling of KDP crystal with the soft-brittle property. The following conclusions can be drawn based on this research:

(1) FFT analysis is a useful way to filter out the noises from the cross feed force and thrust force in micro ball end-milling of KDP crystal, while it is failed to filter the noises from the feed force because the feed force is relatively small which is easily influenced by external environment in the measurement.
(2) The changing trend of roughness value Ra of machined grooves and the static component of the thrust force is similar except for the size effect area. The correlation coefficients between the static component of thrust force and roughness value Ra of machined grooves are larger than 0.97 indicating that the roughness value Ra of machined grooves could be predicted by monitoring the static component of the thrust force. Relatively large spindle speed helps to improve the machined surface roughness.
(3) The dimensional error of machined groove would be predicted by monitoring the P-V value of thrust force. The dimensional error of the machined groove will increase when the spindle speed is small enough (causing brittle cutting) or large enough (reducing cutting stability) and the spindle speed with a moderate value helps to reduce the dimensional error of machined groove.

## Figures and Tables

**Table 1:** The cutting parameters in the experiments.

Spindle Speed *n* (10^4^ r/min)	Depth of Cut *a*_p_ (μm)	Feed per Tooth *f* (μm/z)	Cutting Length l (mm)	Tilt Angle *β* (°)
1, 2, 3, 4, 5, 6, 7	2, 5, 8	1	3	30

**Table 2:** The correlation coefficients between the static component of thrust force and roughness value Ra of machined grooves with spindle speed under different depth of cut.

Depth of Cut (μm)	2	5	8
Correlation Coefficients	0.9964	0.9745	0.9894

**Table 3:** The correlation coefficients between the P-V value of thrust force and dimensional error of machined grooves with spindle speed under different depth of cut.

Depth of Cut (μm)	2	5	8
Correlation Coefficients	0.7272	0.5899	0.5182
